# Pneumatosis cystoides intestinalis: A case report

**DOI:** 10.1097/MD.0000000000028588

**Published:** 2022-01-21

**Authors:** Qiuyu Zhang, Xiangke Niu, Cong Wang, Qiang He, Junying Xiang

**Affiliations:** a Department of Gastroenterology, Affiliated Hospital & Clinical Medical College of Chengdu University, Chengdu, Sichuan, China; b Department of Radiology, Affiliated Hospital & Clinical Medical College of Chengdu University, Chengdu, Sichuan, China.

**Keywords:** endoscopic ultrasound, fine-needle, pneumatosis cystoides intestinalis

## Abstract

**Rationale:**

Pneumatosis cystoides intestinalis (PCI) is a rare condition characterized by multiple gas-filled cysts in the intestinal wall, and can be caused by many conditions.

**Patient concerns:**

We reported a-69-year-old man with a long history of chronic obstructive pulmonary disease was admitted to the gastroenterology department because of alternating bowel movement and intermittent bloody stool.

**Diagnoses:**

Colonoscopy revealed multiple nodular protuberances covered with normal-looking mucosa in the ascending and proximal transverse colon. Abdominal computed tomography scan and endoscopic ultrasound revealed multiple gas-filled cystic lesions in the submucosa. The diagnosis of PCI was confirmed by cyst collapse after puncturing with a fine needle.

**Interventions and outcomes:**

Considering that the patient had no peritonitis or other complications, conservative approaches, including oxygen inhalation and oral probiotics, were used. The patient was transferred to the anorectal department after 5days of clinical observation in good condition to further treat hemorrhoids.

**Lessons:**

PCI is a rare condition that may be secondary to many other diseases. Because of its atypical clinical manifestations, it can be misdiagnosed as other diseases, such as polyps, inflammatory bowel disease, and even cancer. The diagnosis of PCI depends on computed tomography, colonoscopy, and endoscopic ultrasonography. Fine-needle aspiration may be helpful in the diagnosis and treatment of PCI.

## 1 Introduction

Pneumatosis cystoides intestinalis (PCI) is an uncommon condition characterized by the presence of gas-filled cysts in the intestinal wall. PCI was first described by Duo Vernoi in autopsy specimens in 1730.^[1]^ The causes of PCI vary, 85% of which are secondary to other conditions such as abdominal trauma, intestinal obstruction, inflammatory bowel disease, malignant tumor, chemoradiotherapy, chronic lung diseases, and connective tissue diseases (CTD).^[2–4]^ Diagnosis is challenging because of the asymptomatic or atypical symptoms. Diagnosis mainly depends on the characteristic imaging manifestations. Some studies have indicated that endoscopic ultrasound (EUS) and endoscopic fine-needle aspiration are useful for the diagnosis and treatment of PCI.^[5–8]^ Here, we report the case of a 69-year-old man admitted to the gastroenterology department with alternating bowel movements and intermittent bloody stool. PCI was diagnosed using abdominal computed tomography (CT) scan, colonoscopy, and EUS. Fine-needle aspiration is helpful for the diagnosis and treatment of PCI.

## 2 Case presentation

**Figure 1:**
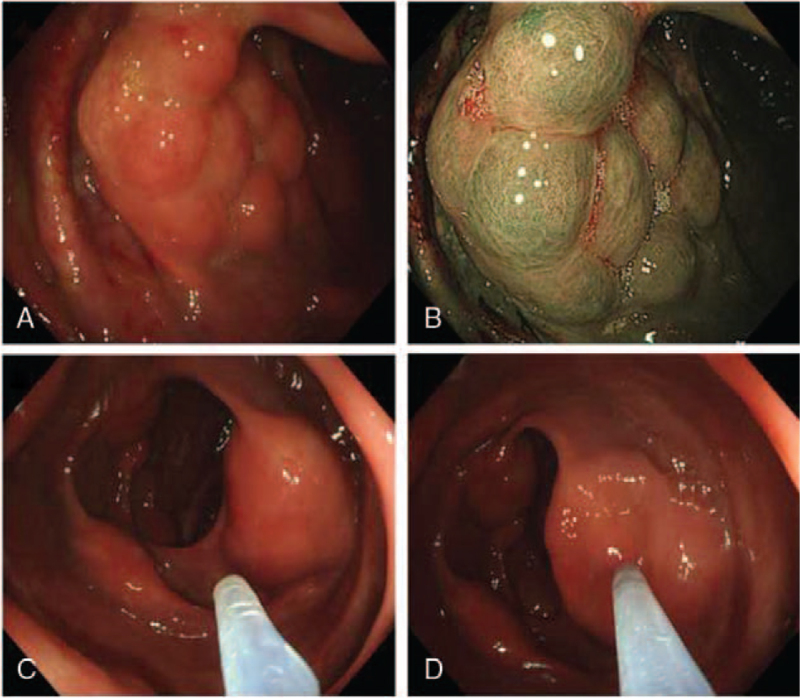
Colonoscopy image of pneumatosis cystoides intestinalis. A, B: Colonoscopy showing nodular protuberances covered with normal-looking mucosa in the ascending colon. C, D: The cyst collapse after puncturing the cyst.

**Figure 2:**
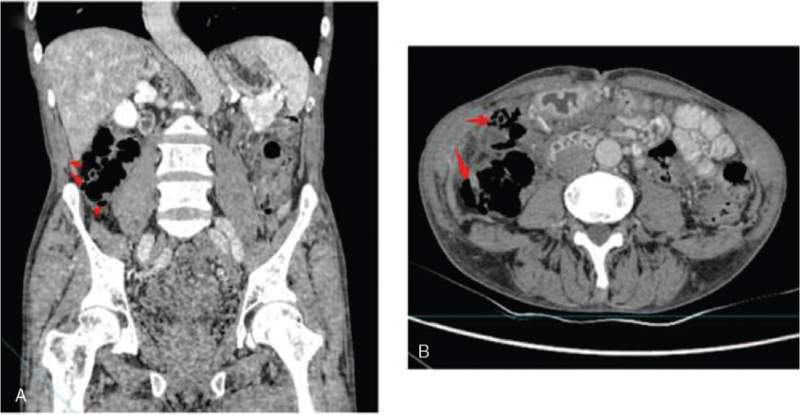
Coronal plus axial abdomen CT showing the intramural gas in the ascending colon.

**Figure 3:**
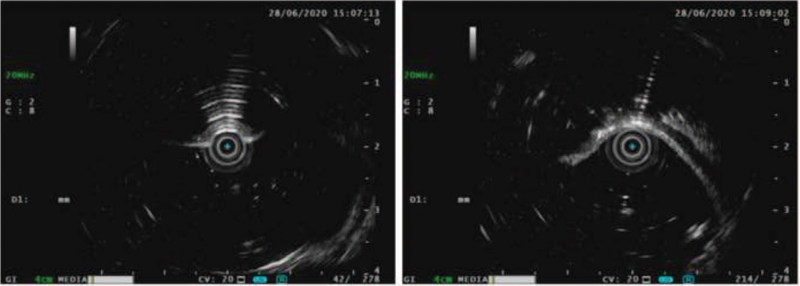
Endoscopic ultrasound with 20 MHz-probe showing hyperechoic air pockets in the submucosa of the ascending colon with ringing artifacts.

A 69-year-old man was admitted to the gastroenterology department of our hospital because of alternating bowel movements and intermittent bloody stools for 2 years. In the past 2 years, his weight had decreased by approximately 25 kg, and the patient had no obvious abdominal pain, abdominal distension, vomiting, or diarrhea. He lived in the plateau area of Songpan County, Aba Tibetan and Qiang Autonomous Prefecture, Sichuan Province. He had a history of irregularly treated chronic obstructive pulmonary disease (COPD) for 20 years, which was being treated irregularly. A physical examination revealed no abdominal tenderness or distention. Laboratory tests revealed increases in hemoglobin (207 g/L, normal: 120–160 g/L) and red blood cell count (6.58 × 10^12^/L, normal: 3.80–5.10 × 10^12^/L), and a decrease in white blood cell count (3.17 × 10^9^/L, normal 4–10 × 10^9^ /L). Stool evaluation was normal. Colonoscopy revealed multiple nodular protuberances covered with normal-looking mucosa in the ascending and proximal transverse colon (Fig. 1A, B). Furthermore, EUS revealed multiple gas-filled cystic lesions in the submucosa with characteristic ringing artifacts (Fig. 3). Abdominal CT revealed intramural gas in the ascending colon (Fig. 2). These findings were consistent with the diagnosis of PCI. To further clarify the diagnosis of PCI, we used a mucosal injection needle to puncture the cyst. The cysts collapsed, and no return of blood was observed after puncturing the cysts (Fig. 1C, D). These findings further confirm the diagnosis of PCI. Considering that the patient had no peritonitis or other complications, conservative approaches including oxygen inhalation and oral probiotics were used. The patient was transferred to the anorectal department after 5 days of clinical observation in good condition to further treat hemorrhoids.

## 3 Discussion

PCI is a rare condition characterized by multiple gas-filled cysts in the intestinal wall. It was first described by Duo Vernoi in autopsy specimens in 1730^[**Error! Bookmark not defined.**]^. The symptoms of PCI vary and may be asymptomatic or atypical, such as abdominal pain, abdominal distension, nausea, vomiting, and bloody stool.

As previously mentioned, PCI can be primary or secondary to other diseases, and the secondary type accounts for 85% of the cases. However, the mechanism through which gas enters the intestinal wall is not well understood. Multiple hypotheses have been proposed to explain this mechanism. Among them, the three most well-accepted hypotheses are the pulmonary, mechanical, and bacterial theories.^[7]^ Pulmonary theory suggests that chronic lung diseases, such as COPD and asthma, may rupture alveoli, causing mediastinal emphysema and trapping gas into the intestinal wall along the aorta and mesenteric vessels. The mechanical theory refers to the increased intraluminal pressure caused by intestinal obstruction or other diseases that can lead to mucosal damage and promote cyst formation. The bacterial theory refers to the intestinal bacteria that produce gas and trap the gas into the submucosa^[9]^ or aerogenic bacteria that directly penetrate the intestinal mucosa into the submucosa and produce gas.^[10]^

Our patient suffered from a long history of COPD which was supposed to be related to the formation of gas-filled cysts in the colon. In addition, he lived in a high-altitude area, where the oxygen partial pressure decreased. Chronic hypoxia in the plateau can cause hypoxic pulmonary vascular remodeling and subsequent hypoxic pulmonary arterial hypertension, which may lead to the aggravation of chronic lung disease. However, the relationship between high-altitude hypoxia and PCI remains unclear.

Because of non-specific symptoms, laboratory examinations, and pathological characteristics, the diagnosis of PCI mainly depends on abdominal CT scan, radiography, colonoscopy, and EUS. As reported,^[11]^ abdominal CT and colonoscopy are useful tools for the diagnosis of PCI, but they both have their own advantages and disadvantages. CT allows the detection of air-filled cysts in the intestinal wall and the presence of gas in the portal vein or mediastinum. However, CT scan cannot accurately determine intestinal ischemia and necrosis, which are emergency procedures for PCI and indications for urgent surgery. The inability to confirm submucosal lesions and detect subserous pneumatosis are limitations of colonoscopy in the diagnosis of PCI. EUS is reported to be helpful in confirming the diagnosis of PCI by using a miniprobe to detect submucosal lesions in the colonic wall and provide reliable imaging with echoless following the ringing phenomenon.^[5–7,11]^ Hence, EUS is considered the first choice for the diagnosis of PCI under certain conditions. Moreover, endoscopic fine-needle aspiration may be useful for the diagnosis and treatment of PCI. Similar to our patient, when puncturing the cyst, the cyst collapsed after the air escaped.^[8]^

The choice of treatment depends on the complications and the underlying causes of PCI. Most cases are considered benign, and only conservative treatments are needed, including oxygen administration, antibiotic therapy, and fluid administration.^[7,9,12,13]^ A previous study^[14]^ showed that HBOT was the optimal method for patients without threatening complications. However, if the patient suffers from surgical complications such as intestinal obstruction, intestinal perforation, bleeding, intestinal ischemia and necrosis, or the presence of gas in the portal vein, surgical treatment is recommended.^[15–17]^

In conclusion, our patient had COPD for more than 20 years and lived in a plateau area. It is not difficult to diagnose PCI based on the features of abdominal CT, colonoscopy, and EUS. Furthermore, the diagnosis of PCI was confirmed by collapse of the cysts after puncturing with a fine needle. Because there were no serious complications, the patient was discharged after five days of conservative treatment.

## Author contributions

QYZ contributed to the acquisition of patient information and manuscript writing. XK N performed a computed tomography (CT) scan of the abdomen, and CW and QH participated in the diagnosis and treatment of the patient. JY X revised the manuscript and all authors read and approved the final manuscript.

**Investigation:** Xiangke Niu, Cong Wang, Qiang He.

**Supervision:** Junying Xiang.

**Writing – original draft:** Qiuyu Zhang.

**Writing – review & editing:** Qiuyu Zhang.
